# A Single Dose of The Mango Leaf Extract Zynamite^®^ in Combination with Quercetin Enhances Peak Power Output During Repeated Sprint Exercise in Men and Women

**DOI:** 10.3390/nu11112592

**Published:** 2019-10-28

**Authors:** Miriam Gelabert-Rebato, Marcos Martin-Rincon, Victor Galvan-Alvarez, Angel Gallego-Selles, Miriam Martinez-Canton, Tanausú Vega-Morales, Julia C. Wiebe, Constanza Fernandez-del Castillo, Elizabeth Castilla-Hernandez, Oriana Diaz-Tiberio, Jose A. L. Calbet

**Affiliations:** 1 Department of Physical Education and Research Institute of Biomedical and Health Sciences (IUIBS), University of Las Palmas de Gran Canaria, Campus Universitario de Tafira s/n, 35017 Las Palmas de Gran Canaria, Spain; miriamgela@hotmail.com (M.G.-R.); marcos.martinrincon@gmail.com (M.M.-R.); victor_galvan@hotmail.es (V.G.-A.); angelgallegoselles@hotmail.com (A.G.-S.); martinezcantonmiriam@gmail.com (M.M.-C.); orianad10@gmail.com (O.D.-T.); 2 Nektium Pharma, Agüimes, 35118 Las Palmas de Gran Canaria, Spain; tvega@nektium.com (T.V.-M.); jwiebe@nektium.com (J.C.W.)

**Keywords:** ergogenic aids, polyphenols, high-intensity exercise, ischemia, reperfusion, sports nutrition, metabolism, oxygen extraction, RONS, oxidative stress

## Abstract

The mango leaf extract rich in mangiferin Zynamite^®^ improves exercise performance when combined with luteolin or quercetin ingested at least 48 h prior to exercise. To determine whether a single dose of Zynamite^®^ administered 1 h before exercise increases repeated-sprint performance, 20 men and 20 women who were physically active were randomly assigned to three treatments following a double-blind cross-over counterbalanced design. Treatment A, 140 mg of Zynamite^®^, 140 mg of quercetin, 147.7 mg of maltodextrin, and 420 mg of sunflower lecithin; Treatment B, 140 mg of Zynamite^®^, 140 mg of quercetin, and 2126 mg of maltodextrin and Treatment C, 2548 mg of maltodextrin (placebo). Subjects performed three Wingate tests interspaced by 4 min and a final 15 s sprint after ischemia. Treatments A and B improved peak power output during the first three Wingates by 2.8% and 3.8%, respectively (treatment x sprint interaction, *p* = 0.01). Vastus Lateralis oxygenation (NIRS) was reduced, indicating higher O_2_ extraction (treatment × sprint interaction, *p* = 0.01). Improved O_2_ extraction was observed in the sprints after ischemia (*p* = 0.008; placebo vs. mean of treatments A and B). Blood lactate concentration was 5.9% lower after the ingestion of Zynamite® with quercetin in men (treatment by sex interaction, *p* = 0.049). There was a higher Vastus Lateralis O_2_ extraction during 60 s ischemia with polyphenols (treatment effect, *p* = 0.03), due to the greater muscle VO_2_ in men (*p* = 0.001). In conclusion, a single dose of Zynamite^®^ combined with quercetin one hour before exercise improves repeated-sprint performance and muscle O_2_ extraction and mitochondrial O_2._ consumption during ischemia. No advantage was obtained from the addition of phospholipids.

## 1. Introduction

Zynamite^®^, a mango leaf extract rich in the natural polyphenol mangiferin, increases exercise performance when given in combination with luteolin or quercetin [1,2]. Besides, both combinations of polyphenols enhanced the contractile response of human skeletal muscle to ischemia-reperfusion. Since the combination of Zynamite^®^ with quercetin was administered for 48 h to 15 days before the exercise [1,2], it remains unknown whether a single dose of Zynamite^®^ would also have ergogenic effects. 

During intense exercise, fatigue may be caused by a mismatch between oxygen delivery and utilization leading to faster consumption of glycogen stores and activation of the anaerobic metabolism, resulting in H^+^ and P_i_ accumulation, and increased generation of reactive oxygen and nitrogen species (RONS) [3,4,5]. Increased levels of H^+^, P_i_, and RONS may reduce Ca^2+^ release from the sarcoendoplasmic reticulum [6] and diminish troponin calcium sensitivity, diminishing peak power [7,8]. Exercise performance also depends on the capacity of the nervous system to provide an adequate activation signal for the prescribed task [9]. The central nervous drive is, in turn, modulated by sensory feedback from group III/IV afferents acting at spinal and supraspinal levels [10]. This is further compounded by the fact that perceived fatigability depends on many factors including core temperature, hydration, brain oxygenation, blood glucose, and several psychological factors (arousal, executive function, expectations, mood, motivation, pain, and performance feedback) [9].

The polyphenolic combination of Zynamite^®^ with quercetin can counteract fatigue by several mechanisms [1,2]. It has been shown that intake of Zynamite^®^ combined with quercetin during the 48 h preceding repeated-sprint exercise attenuates the decline in brain oxygenation normally seen during prolonged sprinting [11], while it facilitates muscle O_2_ extraction [1,2]. In addition, natural polyphenols and antioxidants could attenuate sensitive afferent signals generated by group III and IV ergoreceptors [7,8], facilitating muscle activation [12].

The main constituent of Zynamite^®^ is mangiferin (2-βd-glucopyranosyl-1,3,6,7-tetrahydroxyxanthone), a xanthone (non-flavonoid polyphenol) abundant in Mangifera indica (mango) leaves, bark and pulp, but also in other edible plants [13]. Mangiferin is a potent antioxidant with iron-chelating properties, exceptionally efficient in protecting against the production of free radicals by the Fenton reaction. The mitochondria and the superoxide producing enzymes (xanthine oxidase (XO) and nicotinamide adenine dinucleotide phosphate-oxidase (NADPH oxidase or (NOX)) are the primary sources of RONS during sprint exercise [14]. Mangiferin could attenuate RONS production by inhibiting XO [15] and thus maintain calcium sensitivity [7] during sprint exercise, which can contribute to preserve or enhance peak power output during high-intensity exercise.

Quercetin is categorized as a flavonol, a flavonoid subclass highly abundant in a variety fruits and vegetables. This plant metabolite may have ergogenic effects during prolonged exercise [16,17,18], and during sprint exercise when given in combination with Zynamite^®^ [1]. Quercetin attenuates the damage caused by ischemia-reperfusion in animal models [19], and in combination with Zynamite^®^ improves contractile muscle function after ischemia-reperfusion [1]. However, these effects were observed after repeated pre-exercise supplementation [1,2]. Quercetin also has XO and NOX inhibiting properties [20,21], which could also contribute to enhancing exercise performance during sprint exercise. It has been reported that the intestinal absorption and oral bioavailability of quercetin may be improved by lipidic vehicles [22].

Therefore, the purpose of this study was to determine whether a single dose of Zynamite^®^ administered in combination with a small amount of quercetin (140 mg), or with quercetin combined with sunflower lecithin, increases exercise performance during repeated-sprint exercise. We hypothesized that a single dose of the combination of Zynamite^®^ with quercetin will improve muscle contractile capacity and muscle oxygenation during repeated sprint exercise to exhaustion. We also hypothesized that these effects will be further augmented by adding sunflower phospholipids to the Zynamite®-quercetin mixture.

## 2. Materials and Methods 

### 2.1. Subjects

In total, 50 subjects (all of them physically active) volunteered to participate in this study (24 women and 26 men) (Table 1). From this pool of volunteers, men and women were recruited randomly to a final experimental group of 40 subjects matched for age and sex. Subjects were accepted in the participation list if they fulfilled the inclusion criteria for the study, i.e., age from 18 to 45 years old; without chronic diseases or recent surgery; non-smoker; normal resting electrocardiogram; body mass index (BMI) below 30 and above 18; no history of disease requiring medical treatments lasting more than 15 days during the preceding 6 months; no medical contraindications to exercise testing and lack of food allergies. All volunteers applying met the inclusion criteria, except for one girl having asthma. Ten subjects were excluded due to medical reasons (five subjects) or time availability (five subjects). All volunteers received oral and written information about the experiments and possible risks before they signed their written consent to participate. The study was performed according to the Declaration of Helsinki and approved by the Ethical Committee of the University of Las Palmas de Gran Canaria (CEIH-2019-02). 

A sample size between 20 and 28 participants was required to provide adequate power to detect an improvement between 5 and 6% in peak power output (α = 0.05, β = 0.80; G *Power v 3.1.9.2) in sprint performance. Nevertheless, a total of 40 subjects were included (20 men and 20 women) to increase statistical power and account for potential dropouts and missing values.

### 2.2. General Overview

Subjects were first familiarized with the equipment and experimental protocol with two familiarization visits to perform submaximal and maximal (sprint) tests on the cycle ergometer. In subsequent days, the pre-tests were carried out to determine their body composition, maximal oxygen uptake (VO_2_max) and anaerobic capacity (accumulated oxygen deficit). At least one week after the last pre-test visit, the main experiments started, each including four maximal sprints. The minimum wash-out period between the main trials was ten days. Subjects were requested to maintain their usual level of physical activity between treatments to a maximum of two sessions per week of no more than 30 min per session. All subjects were requested not to exercise and to refrain from carbonated, caffeinated and alcohol-containing beverages during the 48 h period preceding the main sprint experiments and the 24 h before pre-tests. Subjects were also instructed to refrain from taking drugs, medications, dietary supplements and the use of any putative recovery treatments during the duration of the study. 

### 2.3. Pre-Tests

Body composition was determined by dual-energy X-ray absorptiometry (Lunar iDXA, GE Healthcare, WI, USA) as described elsewhere [23]. Subjects performed a familiarization visit during which incremental exercise to exhaustion and an all-out sprint were performed. After familiarization, subjects reported to the laboratory to complete different tests on separate days. First, their VO_2_max, maximal heart rate (HRmax) and maximal power output (Wmax) were determined in normoxia (F_I_O_2_: 0.21, P_I_O_2_: 143 mmHg) with an incremental exercise test to exhaustion with verification [24]. The incremental exercise test started with three min at 20 W, followed by 15 W and 20 W increases every three minutes in women and men, respectively, until the respiratory exchange ratio (RER) was ≥ 1.0. After completion of the load eliciting a RER ≥ 1.0, the intensity was increased by 10 and 15 W/min (women and men, respectively) until subject reached their limit of tolerance (exhaustion). The intensity attained at exhaustion was taken as the maximal power output of the incremental exercise test (Wmax). At exhaustion, the ergometer was unloaded, and subjects remained seated on the cycle ergometer pedaling at a low cadence (30–40 rpm) for 3 min. This was followed by the verification test starting at Wmax + 5 W for 1 min, with increases of 4 and 5 W (women and men, respectively) every 20 s until exhaustion. During the incremental tests to exhaustion and the main sprint experiments, gas exchange was continuously recorded using a Vyntus CPX (Jaeger-Carefusion, Hoechberg, Germany) metabolic cart in breath-by-breath mode. The metabolic cart was calibrated prior to each test following manufacturer instructions with high-grade calibration gases provided by the manufacturer of Vyntus CPX. Respiratory variables were averaged every 20 s during the incremental test and the highest averaged value was reported as the VO_2_max.

### 2.4. Main Sprint Experiments

The volunteers were randomly assigned to three different treatments (A, B and C) which were administered in a single dose 1 h before the repeated-sprint protocol, following a double-blind cross-over and counterbalanced experimental design. Treatment A consisted of 140 mg of Zynamite^®^ (standardized to 60% mangiferin), 140 mg of quercetin (in the form of 280 mg *Sophora japonica* extract, standardized to 50% quercetin), 147.7 mg of maltodextrin, and 420 mg of sunflower lecithin. Treatment B consisted of 140 mg of Zynamite^®^, 140 mg of quercetin (in the form of 280 mg *Sophora japonica* extract, standardized to 50% quercetin), and 2126 mg of maltodextrin. Treatment C consisted of 2548 mg of maltodextrin (placebo). All treatments were administered in methylcellulose capsules with identical appearance. 

On the days of the main experiments, subjects reported to the laboratory with a 4 h to 10 h period of fasting, depending on the testing time of the day. Each subject thoroughly recorded the last dinner and the last meal preceding the main experiment. Subjects performed all three trials at the same time of the day (±1 h) and were asked to reproduce precisely the fasting period and the preceding meals for each of the trials. Sixty minutes before the start of the experiment, they ingested the supplement tested with 300 mL of water. During the following 60 min, subjects were instrumented and prepared for the exercise, while their hemoglobin concentration was measured in capillary blood (HemoCue, Ängelholm Sweden). After that, subjects sat on the cycle ergometer and performed a 6 min warm-up pedaling at 80 or 100 W (women and men, respectively) keeping the pedaling cadence at 80 rpm (±3 rpm). 

**Figure 1:**
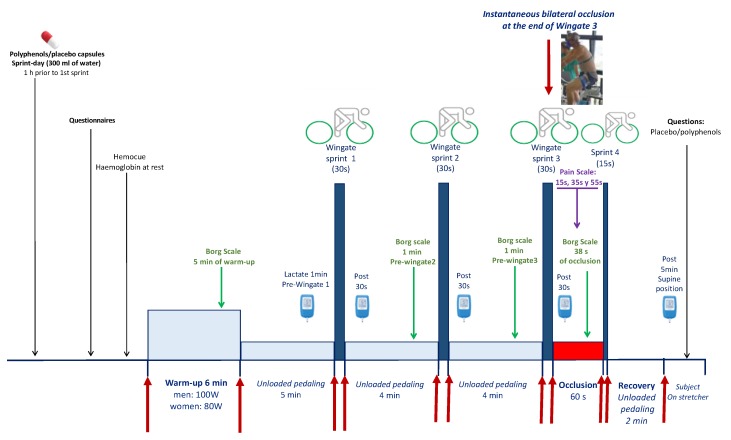
Experimental protocol.

After a 4.5 min period of unloaded pedaling, they stopped pedaling, and the ergometer was switched to isokinetic mode. At the 5^th^ minute, they performed a Wingate test (30 s all-out sprint in isokinetic mode at 80 rpm). This was followed by 3.5 min of unloaded pedaling and another 30 s period, during which they stopped pedaling, and the ergometer was switched to the isokinetic mode. At the 4^th^ minute, a second 30 s Wingate test was performed, which was also followed by another 3.5 min of unloaded pedaling and another 30 s period of rest. A third 30 s Wingate was then performed. Immediately at the end of the third 30 s sprint, the circulation of both lower extremities was instantaneously occluded for 60 s by inflating bilateral cuffs at 300 mmHg as previously reported [25,26] (Figure 1). For this purpose, bilateral cuffs were placed around the thighs during the preparation phase, as close as possible to the inguinal crease, and were connected to a rapid (0.3 s) cuff inflator system (SCD10, Hokanson E20 AG101, Bellevue, WA, USA) before they were seated on the cycle ergometer. 

Fifty seconds after the start of the occlusion, a reverse countdown was started, and the subjects re-started pedaling as fast and hard as possible for 15 s, with the ergometer set in the isokinetic mode. At the beginning of the sprint, the cuff was deflated to allow for a full reestablishment of the circulation during the last sprint. This was followed by two minutes of unloaded pedaling. After that, the subjects rested supine on a laboratory stretcher. One min before the first Wingate test, 30 s after each Wingate test and five min after the end of the final 15 s sprint, a capillary blood sample was drawn from the earlobe, previously hyperhemized with Finalgon^®^ cream, to measure the concentration of lactate (Lactate-Pro 2, Arkray, Kyoto, Japan). Before the start and at the end of the experiments, the subjects were asked whether they could figure out what kind of supplement they had. They were previously informed that they would be ingesting polyphenols in two of the three experiments, and placebo in the remaining experiment (Figure 1).

### 2.5. Power Output

During the incremental exercise protocol (pre-test) subjects were requested to maintain the pedaling rate at 80 rpm (±3 rpm) while the cycle ergometer (Excalibur Sport 925900, Lode, Groningen, The Netherlands) was set in rpm-independent mode. Exhaustion was defined by the incapacity to maintain a pedaling rate above 50 rpm during 5 s, despite strong verbal encouragement, or by a sudden stop in pedaling. For the main sprint experiments, the cycle ergometer was set on isokinetic mode at 80 rpm for all sprints and in an rpm-independent mode during the warm-up and recovery phases. During the isokinetic sprints, the volunteers pedaled as fast and hard as possible, exerting as much force on the pedals as they could at each pedal stroke from the start to the end of the sprint. The servo-control brake system of the cycle ergometer adjusts continuously and almost instantaneously the braking force, so the pedaling rate stays at 80 rpm during the whole sprint. Data from all isokinetic sprints were reported as instantaneous peak power (PPO) and mean power output (MPO). Strong verbal encouragement was provided throughout the entire protocol. 

### 2.6. Oxygen Demand and Deficit 

The O_2_ demand during the supramaximal exercise bouts was estimated from the linear relationship between the averaged VO_2_ for the last minute of each load, from 20–40 W to the highest intensity with an RER < 1.00 in the incremental exercise test. The accumulated oxygen deficit (AOD), representing the difference between O_2_ demand and VO_2_, was determined as previously reported [27].

### 2.7. Vastus Lateralis Muscle and Cerebral Oxygenation

Cerebral oxygenation was assessed at rest and during exercise using near-infrared spectroscopy (NIRS, NIRO-200, Hamamatsu, Japan) employing spatially-resolved spectroscopy to obtain the tissue oxygenation index (TOI) using a path-length correction factor of 5.92 [28]. The NIRS optodes were double-sided taped in the lateral aspect of the right thigh at middle length between the patella and the anterosuperior iliac crest, over the middle portion of the musculus Vastus Lateralis. An additional optode was placed on the right frontoparietal region at 3 cm from the midline and 2–3 cm above the supraorbital crest, to avoid the sagittal and frontal sinus areas [11]. This optode placement examines the tissue oxygenation of the superficial frontal cerebral cortex is recorded. The probes were secured in place by double-sided tape and elastic bandages were used to avoid the entrance of external light and minimize movement artifacts.

### 2.8. Assessment of Pain and Effectiveness of Concealment 

Subjects were requested to rate the level of pain felt during the occlusion from 0 to 10, being 10 the highest muscle pain ever suffered during or after exercise in their life. Likewise, at the end of the experiment, subjects were asked about the kind of supplement they thought they had received to check on the effectiveness of concealment.

### 2.9. Statistical Analysis

Variables were checked for normal distribution by using the Shapiro-Wilks test. A two-way repeated-measures ANOVA was used with two within-subjects factors: treatment (with three levels) and exercise bout (with three levels), and with sex as a between-subjects factor. The Mauchly’s test of sphericity was run before the ANOVA, and in the case of violation of the sphericity assumption, the degrees of freedom were adjusted according to the Huynh and Feldt test. When a significant main effect or interaction was observed, specific pairwise comparisons were carried out with the Least Significant Difference (LSD) post-hoc test. Separate analyses were carried out to test for the effects of treatment in measurements performed during the phases of ischemia and the 15 s post-ischemia sprint. For these purposes, a repeated-measures ANOVA was used with one within-subjects factor: treatment (with three levels), and with sex as a between-subjects factor. Values are reported as the mean ± standard deviation of the mean (unless otherwise stated). *p* ≤ 0.05 was considered significant. Statistical analysis was performed using SPSS v.15.0 for Windows (SPSS Inc., Chicago, IL, USA).

## 3. Results

### 3.1. Effects on Sprint Performance

**Figure 2:**
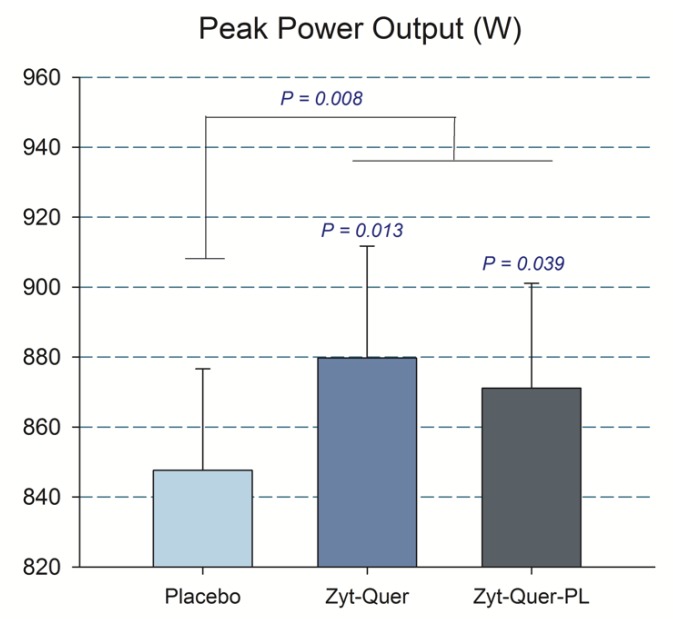
Peak Power output. The bars correspond to the mean of the three sprints, after the ingestion of placebo, Zynamite^®^ (Zyt) combined with quercetin (Quer) or Zynamite^®^ combined with quercetin and phospholipids (PL). Error bars represent the standard error of the mean. *p* values: comparison with placebo. *N* = 40.

**Figure 3:**
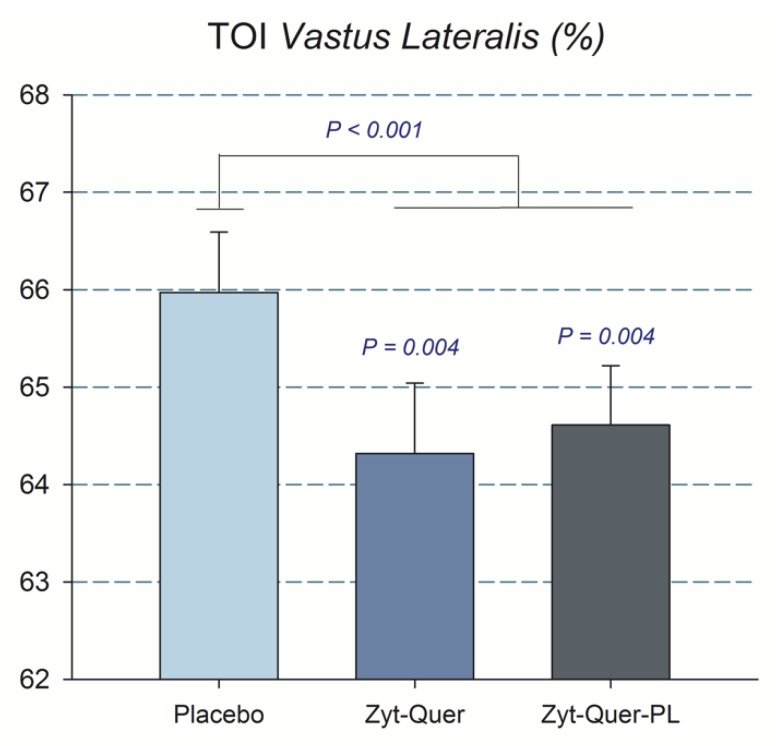
Peak Power output. The bars correspond to the mean of the three sprints, after the ingestion of placebo, Zynamite^®^ (Zyt) combined with quercetin (Quer) or Zynamite^®^ combined with quercetin and phospholipids (PL). Error bars represent the standard error of the mean. *p* values: comparison with placebo. *N* = 40.

Compared to placebo, treatments A and B improved peak power output during the first three sprints by 2.8 (*p* = 0.04) and 3.8% (*p* = 0.01), respectively (ANOVA treatment x sprint interaction, *p* = 0.01) (Table 2) (Figure 2). This effect was accompanied by reduced Vastus Lateralis oxygenation, indicating higher O_2_ extraction (treatment x sprint interaction, *p* = 0.01) (Table 3). This enhanced O_2_ extraction was only observed in men (treatment by sex interaction, *p* = 0.037). Improved O_2_ extraction was also seen in the sprints performed after ischemia (*p* = 0.008; placebo compared with the mean of treatments A and B), due to the effect elicited in men (A vs. B, *p* = 0.04, and A vs. C, *p* = 0.005) (Figure 3). Consequently, a trend to a higher VO_2_peak during the repeated-sprint protocol was observed after the administration of treatment A compared to placebo (+3.7%, *p* = 0.096) and treatment B compared to placebo in men (+1.7%, *p* = 0.065) (Table 4).

**Figure 4:**
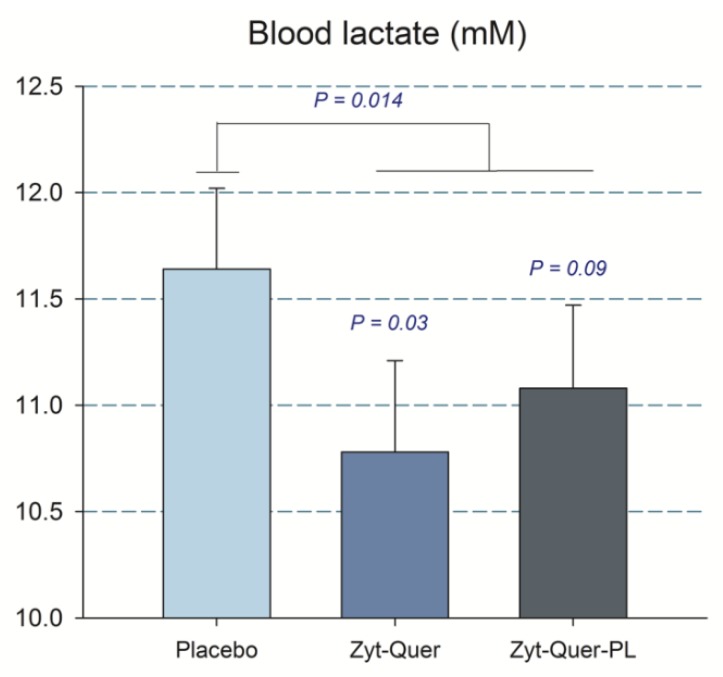
Capillary blood lactate concentration in 20 men. The bars correspond to the mean of the three sprints, after the ingestion of placebo, Zynamite^®^ (Zyt) combined with quercetin (Quer) or Zynamite^®^ combined with quercetin and phospholipids (PL). Treatment by sex interaction for the first three sprints *p* = 0.059. Error bars represent the standard error of the mean. *p* values: comparison with placebo. *N* = 20.

In agreement with a slightly higher reliance on the aerobic metabolism in the sprint performed after the intake of Zynamite^®^ with quercetin, blood lactate concentration tended to be 7.4% lower in men after the administration of treatment B compared to placebo (*p* = 0.03, treatment by sex interaction for the first three sprints, *p* = 0.059) (Figure 4).

**Figure 5:**
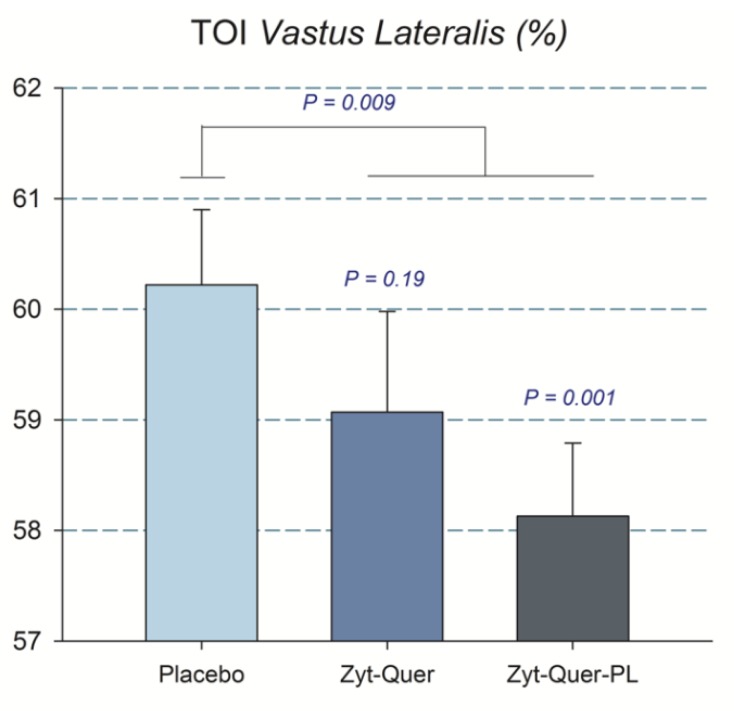
Tissue oxygenation index of the musculus Vastus Lateralis during the 60 s ischemia applied at the end of the third Wingate test. The bars correspond to the mean values after the ingestion of placebo, Zynamite^®^ (Zyt) combined with quercetin (Quer) o Zynamite^®^ combined with quercetin and phospholipids (PL). Error bars represent the standard error of the mean. *p* values: comparison with placebo. *N* = 40.

A similar result was observed when the blood lactate obtained 5 min after the sprint performed following ischemia was included in the ANOVA analysis, i.e., the blood lactate concentration was 5.9% lower after the ingestion of Zynamite^®^ with quercetin (11.9 ± 1.7 and 12.8 ± 1.6 mM, in B and placebo, respectively, *p* = 0.017; treatment by sex interaction, *p* = 0.049). The mean rate of perceived exertion (RPE) during the protocol was similar in the three trials (Table 3) and no significant differences were found in the pain felt at different timepoints nor the mean pain during the occlusion (Table 4). Compared to placebo, during the whole 60 s of ischemia, there was a higher O_2_ extraction by the Vastus Lateralis after the ingestion of polyphenols (ANOVA treatment effect, *p* = 0.03) (Figure 5).

### 3.2. Efficiency of Concealment 

Over a total of 120 tests, in 66 instances subjects reported not to be able to figure out whether they had a placebo or an active polyphenolic substance. In 11 cases of a total of 40 trials with placebo, the subjects guessed correctly when they had been on a placebo.

## 4. Discussion

In agreement with previous reports, Zynamite^®^ enhances peak power output when given in combination with quercetin [1,2]. In the present study, the quercetin dose was about 25–50% lower than that associated with quercetin ergogenic effects in previous studies [16], suggesting that this polyphenolic combination may have synergistic effects. Here, we have shown that even a single dose of Zynamite^®^ combined with quercetin administered one hour before exercise improves repeated-sprint exercise performance and muscle O_2_ extraction. As expected with greater reliance on the aerobic energy production, blood lactate concentration was reduced. We have also confirmed that the combination of Zynamite^®^ with quercetin improves exercise performance and muscle O_2_ extraction capacity in the sprint performed after ischemia. 

Previous studies reporting ergogenic effects after quercetin administration have used supplementation regimes characterized by higher doses (>600 mg), administered during several days [16]. In contrast with our findings, a previous study has reported no effect of supplementation with quercetin (1000 mg/day for one week) on repeated-sprint performance (12 × 30 m maximal effort sprints) in team-sport athletes [29]. Others have concluded that the effect of quercetin in athletes is unclear [18].

### 4.1. The Combination of Zynamite^®^ with Quercetin Improves Peak Power Output During Repeated Maximal Sprints

Sprint exercise elicits high glycolytic rates, lactate accumulation, and reduction of muscle pH [30]. In turn, acidification facilitates the production of hydroxyl radicals by the Fenton reaction and reduces the activities of the antioxidant enzymes glutathione reductase, glutathione peroxidase, and glutathione S-transferase [31]. Quercetin [32,33] and its colonic catecholic metabolites [34] possess potent free radical-scavenging capacity. Mangiferin, the main polyphenol present in mango leaf extract, is also a potent antioxidant [35,36]. In vitro studies indicate that the antioxidant capacity of polyphenol mixtures exceeds that of their constituents [36,37]. However, each polyphenolic compound has unique chemical properties which determine some specific actions in different cellular compartments [38,39]. Although *in vivo* experimental evidence is lacking, a combination of polyphenols likely counteracts more efficiently the RONS produced during exercise in different subcellular compartments of the skeletal muscle fibers than single compounds [40]. 

RONS may reduce Ca^2+^ release from the sarcoendoplasmic reticulum [6] and troponin calcium sensitivity, lowering peak power [7,8]. However, excessive antioxidant capacity may also limit force generation [41]. This could also explain why in the present investigation, an ergogenic effect was seen at a rather small dose of quercetin, which has not been tested previously in humans. The impact of RONS on muscle force follows a bell-shaped curve [42], and therefore, antioxidants at high dose may be detrimental [29]. Nevertheless, it has been reported that 12 weeks of quercetin supplementation at doses of 500 to 1000 mg/day, combined with 125 or 250 mg of vitamin C/day, respectively, had no effect on oxidative stress and antioxidant capacity [43].

An alternate mechanism by which the combination of quercetin and mangiferin may have limited RONS production during exercise is through the inhibition of XO and NOX [15,20,21], which play a crucial role as sources of RONS during sprint exercise [14,44]. Although mangiferin can cross the blood-brain barrier, modulate neurotransmission, K^+^ channels and nociception [45], and attenuate sensory feedback, no significant effects on RPE were observed in the present investigation.

### 4.2. The Combination of Zynamite^®^ with Quercetin Improves O_2_ Extraction and Reduces Peak Blood Lactate Concentration During Repeated Sprint Exercise 

Oxygen extraction depends on muscle oxygen diffusing capacity, oxygen delivery, and the PO_2_ gradient from the muscle capillaries to the mitochondria [46]. Muscle O_2_ diffusing capacity does not limit VO_2_ during 30 s all-out sprints, because there is a large functional reserve in muscle O_2_ diffusing capacity [47]. Muscle blood flow during sprint exercise is determined by cardiac output [47] and vascular conductance [48]. Increasing muscle blood flow may enhance the mean capillary PO_2_ and hence, the gradient for O_2_ diffusion, which could improve O_2_ extraction. At maximal exercise, skeletal muscle vascular conductance is assumed to be maximal. This has been shown by experiments in which no increase of vascular conductance was observed in subjects exercising maximally with the intra-arterial infusion of maximal doses of ATP (one of the most potent vasodilators) [49]. An increase of skeletal muscle blood flow at maximal exercise is unlikely since this requires a higher cardiac output. The mean heart rate during the sprints was almost identical in the three conditions, suggesting unchanged cardiac output after the polyphenol administration. Thus, assuming that muscle O_2_ diffusing capacity does not limit muscle VO_2_ during sprint exercise, and that muscle blood flow was likely similar in the three conditions, the only mechanism that could explain an improvement in muscle O_2_ extraction is an increase of the gradient driving diffusion. This gradient may be increased by reducing the mitochondrial P_50_ [50] or by improving mitochondrial bioenergetics facilitating a higher muscle VO_2_. 

The mitochondrial respiratory rate and ATP production depend, among other factors, on the mitochondrial concentrations of ADP [51] and Ca^2+^ [52,53]. Flavonoids may increase mitochondrial Ca^2+^ concentration by acting on the mitochondrial Ca^2+^ uniporter [54]. Mangiferin improves skeletal muscle mitochondrial ATP production and upregulates several enzymes of the glycolysis, facilitating a higher glycolytic rate in rodent skeletal muscle [55]. Moreover, cell experiments have shown that mangiferin reduces lactate accumulation by improving pyruvate dehydrogenase activity [56]. During high-intensity exercise hemoglobin [47] and myoglobin [57] deoxygenate, with this effect exacerbated in ischemia [58]. Both deoxyhemoglobin and deoxymyoglobin have nitrite reductase activity resulting in the production of NO from nitrite, and oxidation of heme-Fe^2+^ to heme-Fe^3+^ [59]. This reaction is facilitated by H^+^, which increases during both high-intensity exercise and ischemia [26]. Mangiferin and quercetin could facilitate the nitrite reductase activity of deoxyhemoglobin and deoxymyoglobin by preventing the oxidation of Fe_2_^+^ to Fe^3+^ [60]. The NO released or produced within the muscle fibers can bind to cytochrome c oxidase of the mitochondrial electron transport chain, reducing electron flow and oxygen utilization [59], increasing oxidative phosphorylation efficiency in a redox-sensitive manner by decreasing the slipping in the proton pumps [61]. 

In the present investigation we have shown that supplementation with Zynamite® combined with quercetin was associated with lower muscle oxygenation, and this was accompanied by reduced capillary blood lactate concentration and a trend for a higher peak VO_2_ during the sprints. Moreover, during ischemia, muscle O_2_ was reduced to a larger extent after supplementation with Zynamite^®^ combined with quercetin. Since ischemia was applied instantaneously after three maximal sprints, the biochemical environment is thought to be inhibitory for mitochondrial respiration due to lack of O_2_ and inhibition of mitochondrial respiration by acidosis [26,62]. Nevertheless, a reduction of muscle tissue oxygenation was observed during the ischemia, to a larger extent after the administration of Zynamite^®^ combined with quercetin. This implies that our polyphenol mixture improved mitochondrial bioenergetics during the sprints and ischemia. These findings agree with animal experiments showing that mangiferin protects mitochondrial function from the action of several noxious agents [63,64]. Mangiferin and quercetin supplementation could have improved the match between O_2_ demand and perfusion by facilitating vasodilation [65], and hence, contributing to enhancing O_2_ extraction.

In contrast with our previous studies [1,2] mean power output was not improved in the present investigation. While the study population and the methods applied were essentially similar between the current investigation and our previous studies [1,2], the dose of quercetin and the duration of the supplementation was longer in our previous studies. Thus, repeated administration of Zynamite^®^ combined with quercetin or a larger single dose could elicit even higher ergogenic effects. 

## 5. Conclusions

A single dose of Zynamite^®^ combined with quercetin administered one hour before exercise improves muscular performance and O_2_ extraction. Interestingly, the dose of quercetin used in the present investigation was 25–50% lower than that associated with quercetin ergogenic effects in previous studies, what is compatible with a synergistic or additive effect of this polyphenolic mixture. We have also confirmed that Zynamite^®^ combined with quercetin facilitates mitochondrial O_2_ consumption during ischemia, a situation which is observed during prolonged isometric contractions in many sports disciplines. This effect may have clinical applications worth exploring in future studies. Finally, adding sunflower phospholipids to the Zynamite^®^-quercetin mixture had no additional beneficial effects

## Figures and Tables

**Table 1:** Physical characteristics, body composition and VO_2_max.

	Men			Women			*p*
Age (Years)	23.1	±	2.2	23.5	±	2.9	0.64
Height (cm)	174.4	±	5.6	165.1	±	6.5	0.000
Weight (kg)	73.4	±	9.1	59.5	±	8.0	0.000
Body fat (%)	18.5	±	3.7	27.7	±	4.1	0.000
Fat mass (kg)	13.7	±	3.8	16.7	±	4.4	0.03
Lean mass (kg)	56.6	±	6.3	40.3	±	4.2	0.000
Legs lean mass (kg)	20.2	±	2.7	14.1	±	1.8	0.000
VO_2_max (mL min^−1^)	3189.7	±	525.4	2167.4	±	279.2	0.000
VO_2_max (mL kg^−1^ min^−1^)	43.5	±	5.4	36.6	±	3.9	0.000
LLM VO_2_max (mL kg^−1^ min^−1^)	158.7	±	19.3	154.4	±	17.1	0.47
Hemoglobin (g dL^−1^)	15.4	±	0.7	13.3	±	0.7	0.000
Systolic BP (mmHg)	122.4	±	9.4	111.3	±	5.4	0.000
Diastolic BP (mmHg)	66.2	±	7.3	67.9	±	4.2	0.365

LLM: lower extremities lean mass, also legs lean mass; BP: blood pressure; *N* = 20 for men and women.

**Table 2:** Power output and cardiorespiratory variables during the first three 30 s Wingate tests (mean ± SD).

		A			B			C			Treatment	Treat × Sex	Treat × Wing	Treat × Wing × Sex
PPO (W)	All	871	±	193	880	±	203	848	±	184	0.01	0.048	0.96	0.79
	M	1025	±	133	1015	±	171	981	±	152				
	W	717	±	93	738	±	120	714	±	95				
MPO (W)	All	482	±	107	479	±	109	478	±	109	0.80	0.39	0.70	0.84
	M	570	±	75	564	±	81	567	±	80				
	W	394	±	39	390	±	41	389	±	34				
VO_2_ (mL)	All	1069	±	245	1054	±	228	1064	±	225	0.69	0.24	0.38	0.60
	M	1269	±	177	1226	±	175	1246	±	153				
	W	870	±	88	874	±	103	882	±	104				
VCO_2_ (mL)	All	1102	±	278	1093	±	273	1108	±	276	0.43	0.61	0.28	0.84
	M	1336	±	181	1310	±	177	1340	±	166				
	W	869	±	104	864	±	129	876	±	128				
RER	All	1.03	±	0.06	1.02	±	0.07	1.03	±	0.08	0.20	0.81	0.49	0.45
	M	1.05	±	0.06	1.05	±	0.06	1.08	±	0.06				
	W	1.00	±	0.06	0.99	±	0.07	0.99	±	0.07				
V_E_ (L min^−1^)	All	49	±	12	50	±	12	50	±	13	0.41	0.64	0.14	0.15
	M	57	±	10	58	±	10	58	±	10				
	W	41	±	8	42	±	9	41	±	9				
P_ET_O_2_ (mmHg)	All	119	±	4	119	±	4	119	±	4	0.59	0.53	0.17	0.26
	M	119	±	3	119	±	3	119	±	3				
	W	119	±	4	120	±	4	119	±	4				
P_ET_CO_2_ (mmHg)	All	26	±	3	26	±	4	26	±	3	0.29	0.56	0.12	0.37
	M	27	±	3	27	±	3	27	±	3				
	W	26	±	3	24	±	4	25	±	3				
Heart rate (bpm)	All	163	±	10	165	±	11	164	±	10	0.21	0.31	0.32	0.17
	M	165	±	8	166	±	10	164	±	8				
	W	162	±	12	164	±	12	164	±	11				

M: men; W: women; PPO: peak power output; MPO: mean power output; VO_2_: oxygen uptake; VCO_2_: CO_2_ production; RER: respiratory exchange ratio; V_E_: pulmonary ventilation; P_ET_O_2_: end-tidal O_2_ pressure; P_ET_CO_2_: end-tidal CO_2_ pressure; HR: heart rate. *N* = 39–40 for all variables.

**Table 3:** Oxygen deficit, brain and muscle oxygenation, VO_2_ per watt and rate of perceived exertion for the first three 30 s Wingate tests (mean ± SD).

		A			B			C			Treatment	Treat × Sex	Treat × Wing	Treat × Wing × Sex
Oxygen deficit (mL)	All	1625	±	542	1632	±	622	1612	±	565	0.81	0.82	0.80	0.72
	M	1962	±	556	1982	±	660	1969	±	576				
	W	1289	±	232	1263	±	278	1256	±	238				
Lactate (mM) ^L^	All	11.1	±	1.9	11.3	±	2.0	11.5	±	1.9	0.46	0.059	0.83	0.16
	M	11.1	±	1.8 ^&^	10.8	±	1.9 *	11.6	±	1.7				
	W	11.2	±	2.0	11.9	±	2.1	11.3	±	2.2				
TOI Frontal Lobe (%)	All	66	±	5	65	±	7	67	±	5	0.20	0.62	0.32	0.33
	M	68	±	6	67	±	7	69	±	5				
	W	65	±	3	63	±	6	64	±	4				
TOI Vastus Lateralis (%)	All	64.6	±	3.8	64.3	±	4.5	66.0	±	3.9	0.01	0.04	0.60	0.63
	M	63.4	±	3.7 *	62.3	±	3.9 *	65.2	±	4.5				
	W	65.8	±	3.7	66.5	±	4.1	66.7	±	3.3				
VO_2_/W (mL W^−1^) ^a, L^	All	2.27	±	0.20	2.29	±	0.22	2.29	±	0.19	0.96	0.72	0.73	0.86
	M	2.28	±	0.25	2.28	±	0.26	2.26	±	0.23				
	W	2.25	±	0.12	2.30	±	0.18	2.31	±	0.13				
VO_2_/W/kg LLM (mL W^−1^ kg^−1^) ^b^	All	0.139	±	0.034	0.140	±	0.038	0.141	±	0.035	0.93	0.50	0.42	0.72
	M	0.116	±	0.023	0.114	±	0.027	0.115	±	0.025				
	W	0.162	±	0.026	0.167	±	0.027	0.166	±	0.022				
RPE	All	4.5	±	1.6	4.7	±	1.5	4.7	±	1.5	0.51	0.77	0.57	0.72
	M	4.8	±	1.5	5.0	±	1.4	5.0	±	1.3				
	W	4.2	±	1.7	4.3	±	1.6	4.4	±	1.6				

M: men; W: women; TOI: Tissue oxygenation index; VO_2_: oxygen uptake; LLM: lower extremities lean mass; RPE: rate of perceived exertion. ^a^ Oxygen uptake per watt during the sprints; ^b^ Oxygen uptake per watt and kg of lower extremities lean mass during the sprints; ^L^ analysis done with logarithmically transformed data; * *p* < 0.05 compared to placebo (C); ^&^ 0.05 > *P* < 0.10 compared to placebo. *N* = 39–40 for all variables.

**Table 4:** Peak VO_2_ during the sprints, pain felt during the 60 s occlusions and hemoglobin concentration before the sprints (mean ± SD).

		A			B			C			Treatment	Treat × Sex
Sprints VO_2_peak (mL min^−1^)	All	2668	±	597	2650	±	581	2624	±	566	0.290	0.110
	M	3160	±	391	3099	±	425	3046	±	453		
	W	2177	±	265	2178	±	252	2202	±	279		
Pain 15 s ^L^	All	7.9	±	1.5	8.1	±	1.7	7.6	±	2.0	0.11	0.29
	M	7.6	±	1.6	7.9	±	1.8	7.0	±	2.4		
	W	8.3	±	1.3	8.3	±	1.6	8.2	±	1.5		
Pain 35 s ^L^	All	8.6	±	1.2	8.6	±	1.9	8.4	±	1.7	0.12	0.35
	M	8.5	±	1.3	8.6	±	1.4	8.1	±	2.1		
	W	8.7	±	1.2	8.6	±	2.4	8.8	±	1.2		
Pain 55 s ^L^	All	9.1	±	1.1	9.3	±	1.1	9.2	±	1.0	0.20	0.27
	M	9.0	±	1.2	9.1	±	1.3	9.1	±	1.1		
	W	9.3	±	0.9	9.6	±	0.8	9.3	±	0.9		
Mean Pain ^L^	All	8.5	±	1.2	8.7	±	1.3	8.4	±	1.5	0.12	0.61
	M	8.4	±	1.3	8.5	±	1.4	8.1	±	1.8		
	W	8.7	±	1.1	9.0	±	1.1	8.7	±	1.1		
Hemoglobin (g dL^−1^)	All	14.3	±	1.3	14.4	±	1.3	14.4	±	1.2	0.70	0.09
	M	15.4	±	0.7	15.5	±	0.7	15.3	±	0.6		
	W	13.2	±	0.7	13.3	±	0.8	13.4	±	0.7		

M: men; W: women; Pain 15 s: pain reported at the 15^th^ s of the occlusion; Pain 35 s: pain reported at the 35^th^ s of the occlusion; Pain 55 s: pain reported at the 55^th^ s of the occlusion; ^L^ analysis done with logarithmically transformed data. *N* = 39–40 for all variables.
